# Sequencing and analysis of the gastrula transcriptome of the brittle star *Ophiocoma wendtii*

**DOI:** 10.1186/2041-9139-3-19

**Published:** 2012-09-03

**Authors:** Roy Vaughn, Nancy Garnhart, James R Garey, W Kelley Thomas, Brian T Livingston

**Affiliations:** 1 Department of Cell Biology, Microbiology and Molecular Biology, University of South Florida, 4202 E. Fowler Ave, Tampa, FL 33620, USA; 2 Hubbard Center for Genome Studies, University of New Hampshire, 35 Colovos Rd, Durham, NH 03824, USA; 3 Department of Biological, Sciences, California State University Long Beach, 1250 Bellflower Blvd, Long Beach, CA 90815, USA

**Keywords:** Brittle star, Gene regulatory networks, Evolution, Transcriptome

## Abstract

**Background:**

The gastrula stage represents the point in development at which the three primary germ layers diverge. At this point the gene regulatory networks that specify the germ layers are established and the genes that define the differentiated states of the tissues have begun to be activated. These networks have been well-characterized in sea urchins, but not in other echinoderms. Embryos of the brittle star *Ophiocoma wendtii* share a number of developmental features with sea urchin embryos, including the ingression of mesenchyme cells that give rise to an embryonic skeleton. Notable differences are that no micromeres are formed during cleavage divisions and no pigment cells are formed during development to the pluteus larval stage. More subtle changes in timing of developmental events also occur. To explore the molecular basis for the similarities and differences between these two echinoderms, we have sequenced and characterized the gastrula transcriptome of *O. wendtii*.

**Methods:**

Development of *Ophiocoma wendtii* embryos was characterized and RNA was isolated from the gastrula stage. A transcriptome data base was generated from this RNA and was analyzed using a variety of methods to identify transcripts expressed and to compare those transcripts to those expressed at the gastrula stage in other organisms.

**Results:**

Using existing databases, we identified brittle star transcripts that correspond to 3,385 genes, including 1,863 genes shared with the sea urchin *Strongylocentrotus purpuratus* gastrula transcriptome. We characterized the functional classes of genes present in the transcriptome and compared them to those found in this sea urchin. We then examined those members of the germ-layer specific gene regulatory networks (GRNs) of *S. purpuratus* that are expressed in the *O. wendtii* gastrula. Our results indicate that there is a shared ‘genetic toolkit’ central to the echinoderm gastrula, a key stage in embryonic development, though there are also differences that reflect changes in developmental processes.

**Conclusions:**

The brittle star expresses genes representing all functional classes at the gastrula stage. Brittle stars and sea urchins have comparable numbers of each class of genes and share many of the genes expressed at gastrulation. Examination of the brittle star genes in which sea urchin orthologs are utilized in germ layer specification reveals a relatively higher level of conservation of key regulatory components compared to the overall transcriptome. We also identify genes that were either lost or whose temporal expression has diverged from that of sea urchins.

## Background

Sea urchins (Class Echinoidea) have been used as model organisms in developmental biology for more than a century. Over the last two decades intensive work has led to a fairly detailed understanding of the gene regulatory network (GRN) controlling the differentiation of the embryonic germ layers during development in the species *Strongylocentrotus purpuratus *[1-6]. An initial draft of the *S. purpuratus* genome was completed in 2006 [7] and is now in its third revision [6]. Several expression databases for various embryonic stages have also been constructed using expressed sequence tags (ESTs) [8-11], microarrays [12], and NanoString RNA counting [13]. Here we begin to examine the conservation and divergence in the gene regulatory networks expressed at the gastrula stage in a member of a different echinoderm class, the Ophiuroidea. Our results indicate that, although there are differences that reflect changes in the developmental processes, there is a shared ‘genetic toolkit’ central to the echinoderm gastrula, a key stage in embryonic development.

**Figure 1:**
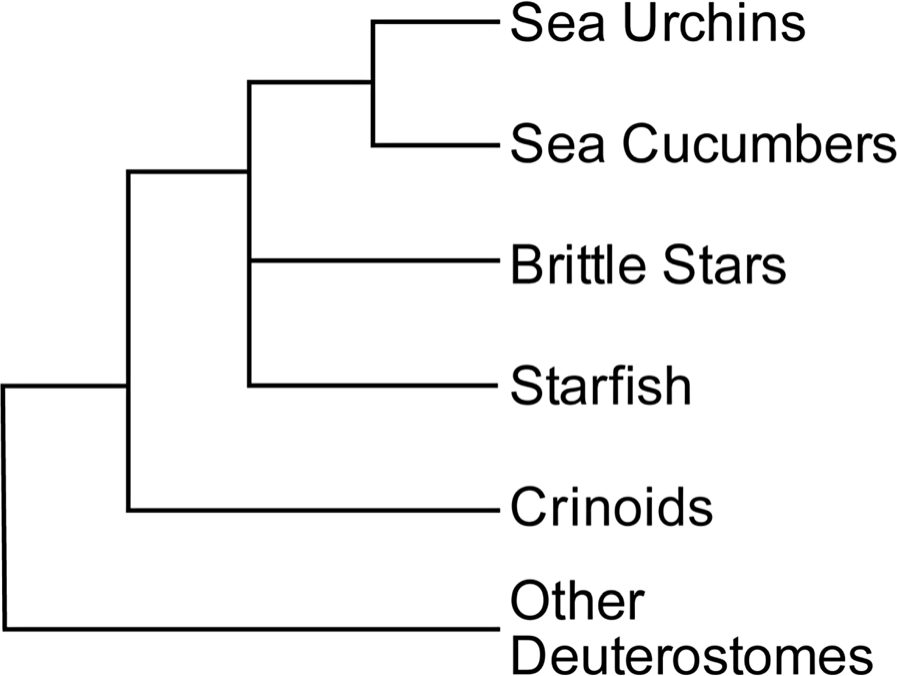
**Phylogeny of echinoderms.** All evidence indicates that crinoids are the most basal. The other four groups all diverged within a very short geological timeframe around 500 million years ago. Urchins and sea cucumbers are generally considered to form a clade of the most derived. It remains unclear whether the brittle stars group more closely with this clade or with starfish, due to conflicts between molecular, morphological, and embryological evidence [14-16].

**Figure 2:**
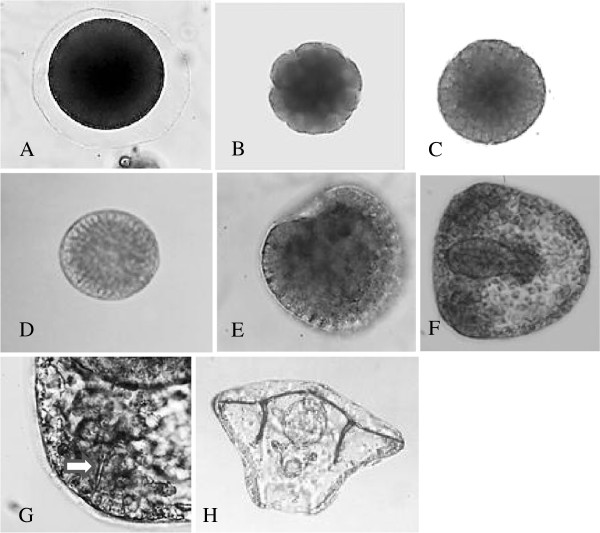
***Ophiocoma wendtii *embryonic development.** Stages (**A**) egg, (**B**) 16 cell (5 h), (**C**) hatched blastula (16 to 18 h), (**D**) mesenchyme blastula (24 h to 26 h), (**E**) early gastrula (28 to 30 h), (**F**) gastrula (38 to 40 h), (**G**) ventrolateral cluster with skeletal spicule (arrow) at 40 h, (**H**) pluteus (80 h).

The echinoderms consist of five living classes: Asteroidea (starfish), Echinoidea (sea urchins and sand dollars), Ophiuroidea (brittle stars) Holothuroidea (sea cucumbers), and Crinoidea (sea lilies and feather stars). The crinoids appear first in the fossil record and are clearly the most basal anatomically. The other four classes appear to have all diverged within a very short geological period around 500 million years ago [14], and the exact phylogenetic relationship of the brittle stars to the other classes remains uncertain due to conflicts between molecular, morphological, and embryological evidence [15,16] (Figure 1). The embryos of all echinoderm classes share some features, including holoblastic cleavage and similar cell movements during gastrulation. However, there are notable differences, such as the formation of micromeres in sea urchins but not brittle stars (Figure 2), the absence of pigment cells in brittle stars, and the formation of an embryonic skeleton in sea urchins and brittle star embryos, but not in the other groups. What is currently unclear is how these similarities and differences in development are reflected in the pattern of gene transcription. Davidson and Erwin [17] have suggested that key gene regulatory subcircuits central to the formation of major morphological features (‘kernels’) are very highly conserved by stabilizing natural selection, both because they are critical to the formation of a complete viable body and because their internal linkages and feedback loops make their component genes mutually dependent. A refinement of this idea is that some of the component transcription factors may be exchanged for others as long as the overall input/output logic and reliability of the circuit and its resulting function are maintained [18]. This suggests that many of the regulatory kernels shown to be important in sea urchin gastrulation would be conserved in the other echinoderm groups.

The set of genes that control skeleton formation in echinoderms may represent such a circuit under evolutionary constraints. All echinoderms form skeletons as adults; however, only sea urchins and brittle stars form extensive embryonic skeletal spicules*.* It has recently been shown that most of the same regulatory genes that underlie skeletogenesis in the sea urchin embryo are also expressed in the construction of the adult skeleton in both sea urchins and starfish [19]. The embryonic skeletons of sea urchins and brittle stars are thus thought to be derived characters resulting from early activation of an adult gene regulatory network in the embryo.

The process of embryonic skeletogenesis has been extensively studied in sea urchins [20]. Asymmetric fourth and fifth cleavages produce four small micromeres and four larger micromeres at the vegetal pole. Descendants of the larger micromeres ingress into the blastocoel just prior to gastrulation and become the primary mesenchyme (PMC), which soon produces the embryonic skeleton. Micromeres are a derived character unique to crown group sea urchins (euechinoids) [21]. Brittle stars and the more basal sea urchin groups form very similar embryonic skeletons from apparently homologous mesenchymal cells without prior unequal cleavages [22], Figure 2.

We have sequenced and characterized the 40-h gastrula transcriptome of the brittle star *Ophiocoma wendtii*. The gastrula stage was chosen because it represents the point in development at which the three primary germ layers diverge, with ingression of mesenchymal cells and invagination of the gut. At this point in sea urchins the gene regulatory networks that specify the germ layers are established and the genes that define the differentiated states of the tissues have begun to be activated. The early gastrula therefore expresses the greatest number and diversity of developmentally important genes. We report here that the brittle star gastrula expresses genes of all functional classes and appears to share many key developmental regulatory components with other echinoderms. Some regulatory genes, as well as genes expressed in differentiated tissues in the sea urchin gastrula, were not expressed in the brittle star gastrula.

## Methods

### Animals and embryos

Brittle stars (*O. wendtii*) were collected from reefs and rubble piles in the shallow waters of Florida Bay near the Keys Marine Laboratory, Long Key, Florida, between April and October. Animals were sorted by sex, with gravid females identified by the presence of swollen purple gonads visible through the bursal slits. Sperm was obtained from two to three males by injection of 1 to 3 mL of 0.5 to 1.0 M KCl. Shedding of eggs from females was induced by a combination of heat and light shock. Animals were placed in containers in the dark with aeration at 30 to 32°C. Periodically the animals were exposed to bright light. Developing embryos were cultured at 25 to 27°C in filtered sea water. RNA was isolated using Trizol (Life Technologies, Carlsbad, CA, USA) following the manufacturer’s protocol.

### Characterization of transcriptome sequences

Sequencing and assembly of contiguous sequences was carried out as described by Meyer *et. al.*[23]. The comparisons of gastrula transcriptomes were followed by an all-by-all BLAST [24] approach where each comparison was databased. These results were then queried for the identification of orthologous genes using a reciprocal best BLAST (RBB) strategy, and for the identification of gene families following the method of Lerat, *et al.*[25] as implemented previously [26,27]. Gene families and singletons were then annotated using Homology Inspector (HomIn) software, a Java program that stores and queries a set of gene families using the database tool db4o for Java version 7.12 [28]. HomIn links gene families with annotation information including Kyoto Encyclopedia of Genes and Genomes (KEGG) Orthology Database (KO) categories [29], Clusters of Orthologous Groups of proteins (COGs) [30,31], Gene Ontology (GO) categories [32], or any other available annotation.

### Search for GRN components

Glean3 predicted protein sequences for genes involved in the *S. purpuratus* developmental gene regulatory network were retrieved from SpBase [6] using the official gene name. These were used as queries to search the brittle star gastrula transcriptome sequences using TBLASTN at default settings. The best hit for each query was then used to search back against both sea urchin protein sequences and GenBank reference proteins using BLASTX. Sea urchin genes which had RBB hits to brittle star with e-values of 1e-9 or better in both directions were designated as present in the brittle star gastrula transcriptome. These sequences can be found in Genbank using accession numbers JX60067 to JX60106.

### Database and analyses

**Figure 3:**
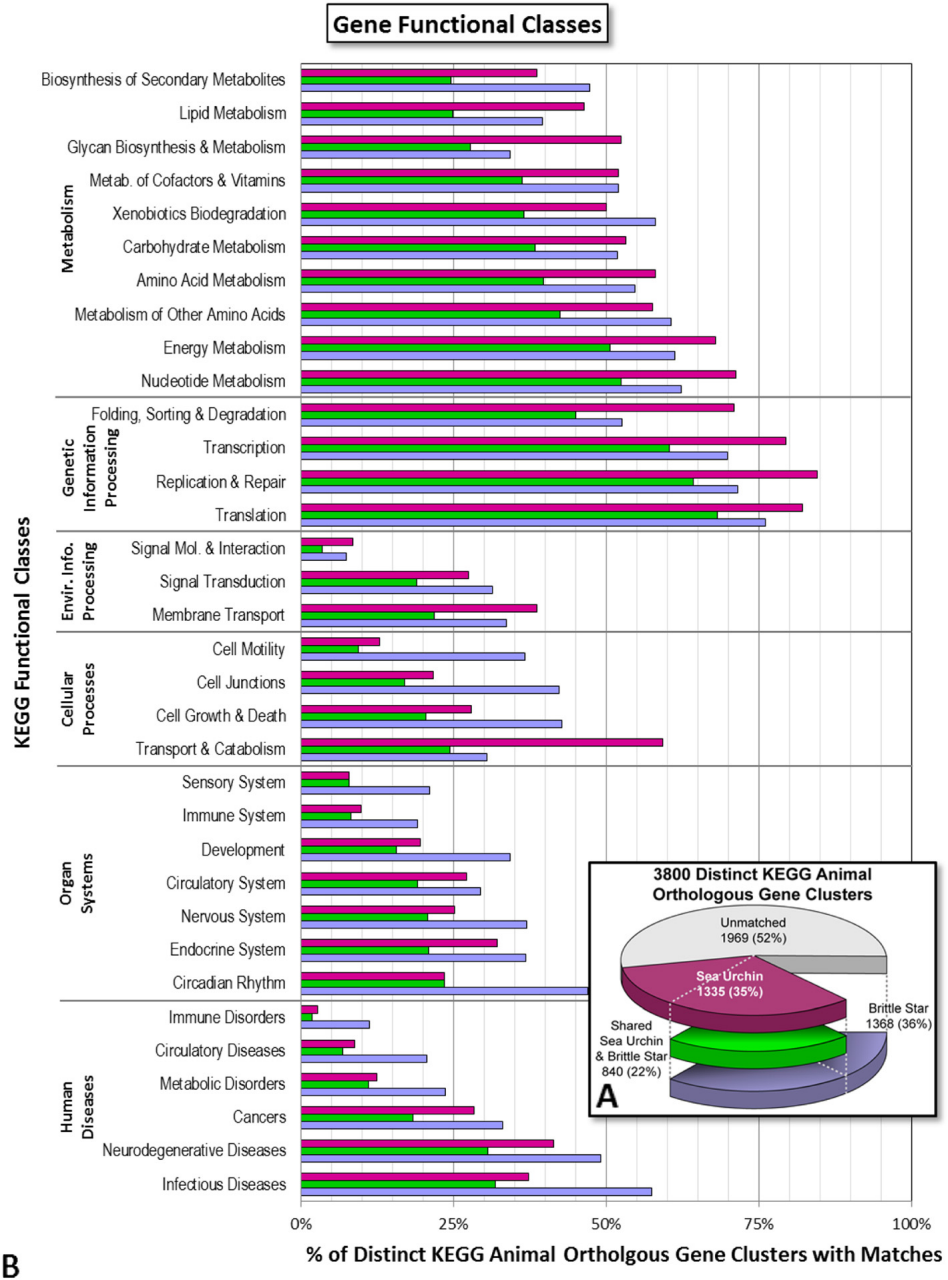
**Gene functional classes found in brittle star gastrula transcriptome.** (**A**) *Ophiocoma wendtii* sequences were compared to the KEGG Orthology database by reciprocal best BLAST. Of 3,800 distinct KEGG animal gene clusters, 36% had significant matches to brittle star (blue), and 35% had matches to sea urchin (purple). Green shows the overlap between these two sets, indicating the KEGG clusters that match to both organisms (22%). (**B**) When sorted into functional classes, an average of 43%, 39%, and 28% of the KEGG clusters within each class had matches to brittle star, to sea urchin, or to both, respectively, with a majority of classes having similar representation in both organisms.

Results from the automated BLAST searches were saved to a Microsoft Access (Microsoft Corporation, Redmond, WA, USA) database. This database and Microsoft Excel (Microsoft Corporation, Redmond, WA, USA) were used for the analyses involving presence/absence of expression, functional classes, and numbers of matches to other databases. Rarefaction curves were generated using EcoSim software [33]. For the functional class analysis, KEGG ortholog clusters were used if they included genes from at least one animal taxon. When a KEGG cluster participates in more than one pathway within a functional class, it was counted only once within the larger functional class. For example, K00128 aldehyde dehydrogenase (NAD+) is part of five different pathways within the class of carbohydrate metabolism and two pathways in lipid metabolism, among many others, but was counted only once within each class in Figure 3B, and once in the total number of distinct KEGG animal clusters in Figure 3A.

## Results and discussion

### Embryonic development of *O. wendtii*

The key stages of *O. wendtii* development are shown in Figure 2. The egg is pigmented, and pigment granules are retained during cleavage stages but disappear in the blastula. Cleavage is radial and holoblastic and is equal throughout cleavage, such that the micromeres characteristic of the sea urchin fourth cleavage division are not produced. A hollow blastula is formed and cells ingress into the blastula to initiate gastrulation. The number of ingressing cells seems much larger than is typical in sea urchins, but we have not quantified the number or traced the lineage of individual cells. Archenteron formation occurs through invagination and convergent extension. A second group of mesenchyme cells forms at the tip of the archenteron and gives rise to the coelomic pouches, but no pigment cells appear. The skeletogenic mesenchyme cells gather in ventrolateral clusters as in sea urchins and begin to form the mineralized skeleton. The timing of development to hatching blastula is similar to sea urchins. However, following the invagination of endoderm, brittle star development proceeds at a slower rate relative to sea urchins. There is an initial invagination at 26 to 30 h post-fertilization, but this persists for several hours before overt endomesoderm development proceeds. Also, unlike that seen in sea urchins, the elongation of the skeletal rods is delayed relative to the extension of the archenteron, such that the archenteron has extended one-third to halfway across the blastocoel before skeletal elements appear. When the gut is fully formed the skeleton is still composed of relatively small tri-radiate spicules. These then elongate such that the pluteus larva is very similar to that of sea urchins. The stage at which we isolated RNA for sequencing analysis is similar to Figure 2F. We chose that point when skeletal elements were first visible.

### Sequencing and assembly

**Figure 4:**
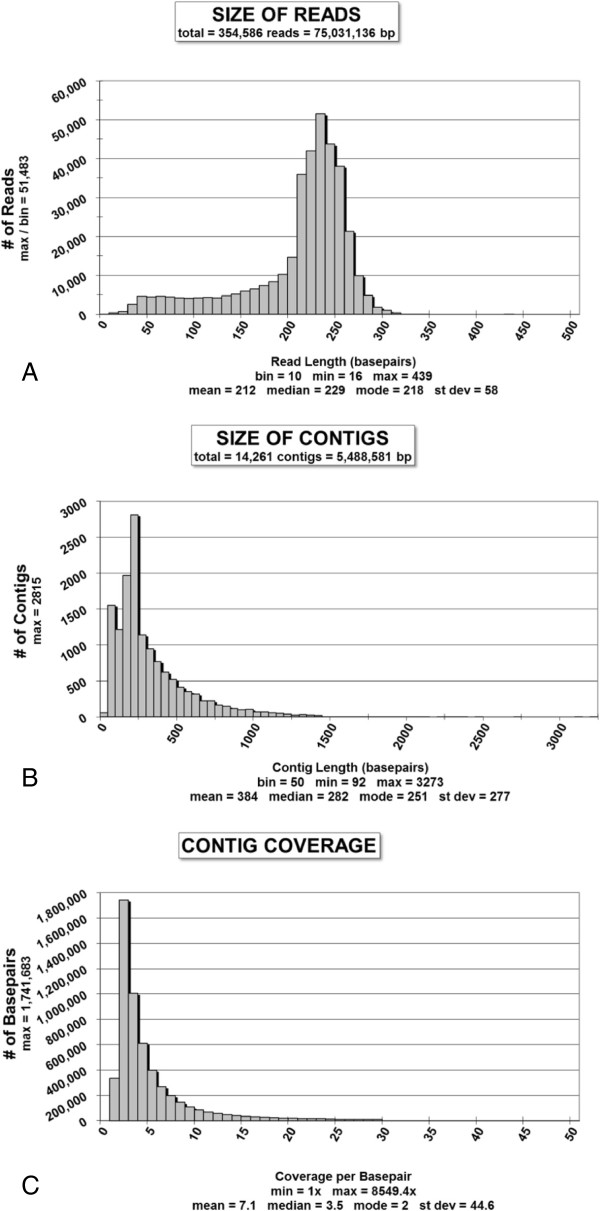
**Pyrosequencing of brittle star transcriptome.** (**A**) After cleaning and trimming, 354,586 reads totaled 75,031,136 bp. Approximately 3/4 had lengths between 200 and 300 bp. Less than one percent were longer than 300 bp. (**B**) A total of 14,261 contigs were assembled, with a combined length of 5,488,581 bp. Median length increased by 23% over that of the unassembled reads. Roughly two-thirds of the reads had lengths between 100 and 400 bp. Four percent were longer than 1,000 bp, creating a long right-hand tail to the distribution. (**C**) The number of times a given nucleotide position is present in the reads used to assemble the contigs ranged from 1x to 8549.4x. Eighty-one percent were represented one to five times, while less than one percent had more than 100× coverage.

Pyrosequencing was performed on mRNA from gastrula stage brittle star embryos. After cleaning and trimming, there were 354,586 sequencing reads with a total of 75,031,136 bp(Figure 4A). Sequencing read lengths ranged from 16 to 439 bp, with approximately three-fourths of the reads being between 200 and 300 bp. Less than one percent were longer than 300 bp. Reads of 15 bp or shorter after trimming were not used for contig assembly. A total of 14,261 contigs were assembled, with a combined length of 5,488,581 bp (Figure 4B). Median length increased by 23% over that of the unassembled reads (282 vs. 229), while average length increased by 81% (384 vs. 212). Roughly two-thirds had lengths between 100 and 400 bp. The average number of reads per contig was 16.3, with a median of 5, a mode of 2, and a maximum of 8,989. Coverage or depth ranged from 1x to 8549.4x, with an average of 7.1, median of 3.5, and standard deviation of 44.6 (Figure 4C).

### Automated annotation

**Figure 5:**
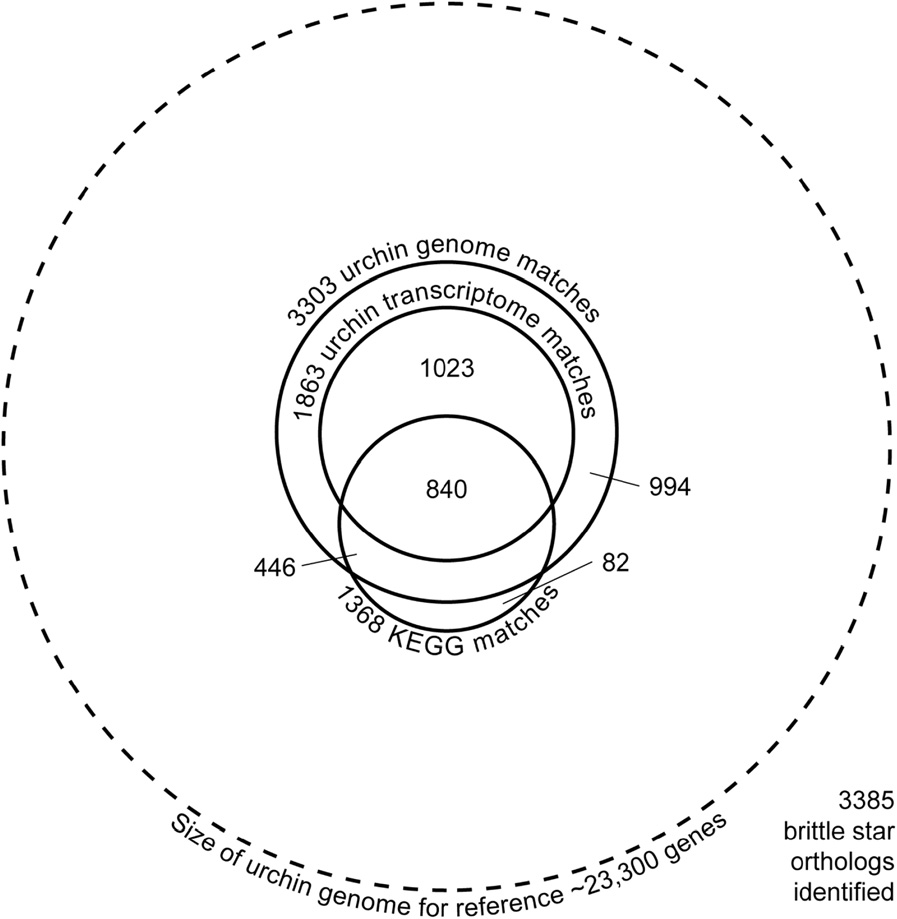
**BLAST identification of brittle star genes.** Automated BLAST was used to align *Ophiocoma wendtii* cDNA sequences to both the genome and transcriptome of the sea urchin *Strongylocentrotus purpuratus*, as well as to the KEGG Orthology database. The areas of the smaller circles represent the number of significant reciprocal best BLAST hits to the indicated datasets. Overlaps indicate matches of the same brittle star sequences to more than one dataset, and in nearly all such cases the matches from the different datasets are mutually consistent. For reference, the large dashed border represents the size of the *S. purpuratus* genome (~23,300 genes).

Reciprocal best BLAST (RBB) searches identified brittle star transcripts putatively corresponding to a total of 3,385 orthologous genes in other databases (Figure 5). The brittle star sequences were translated in all six reading frames, and blastp was used to query the SpBase sea urchin Glean3 protein models. There were 3,303 matches between brittle star and the sea urchin genome [7]. Of these, 1,863 also matched to the sea urchin combined UniGene transcriptome libraries [34]. The KEGG Orthology database [35] produced 1,368 matches. More than two-thirds (2,309 or 68%) of the identified brittle star genes had matches to more than one dataset. Almost a quarter (840 or 24.8%) matched to all three.

**Figure 6:**
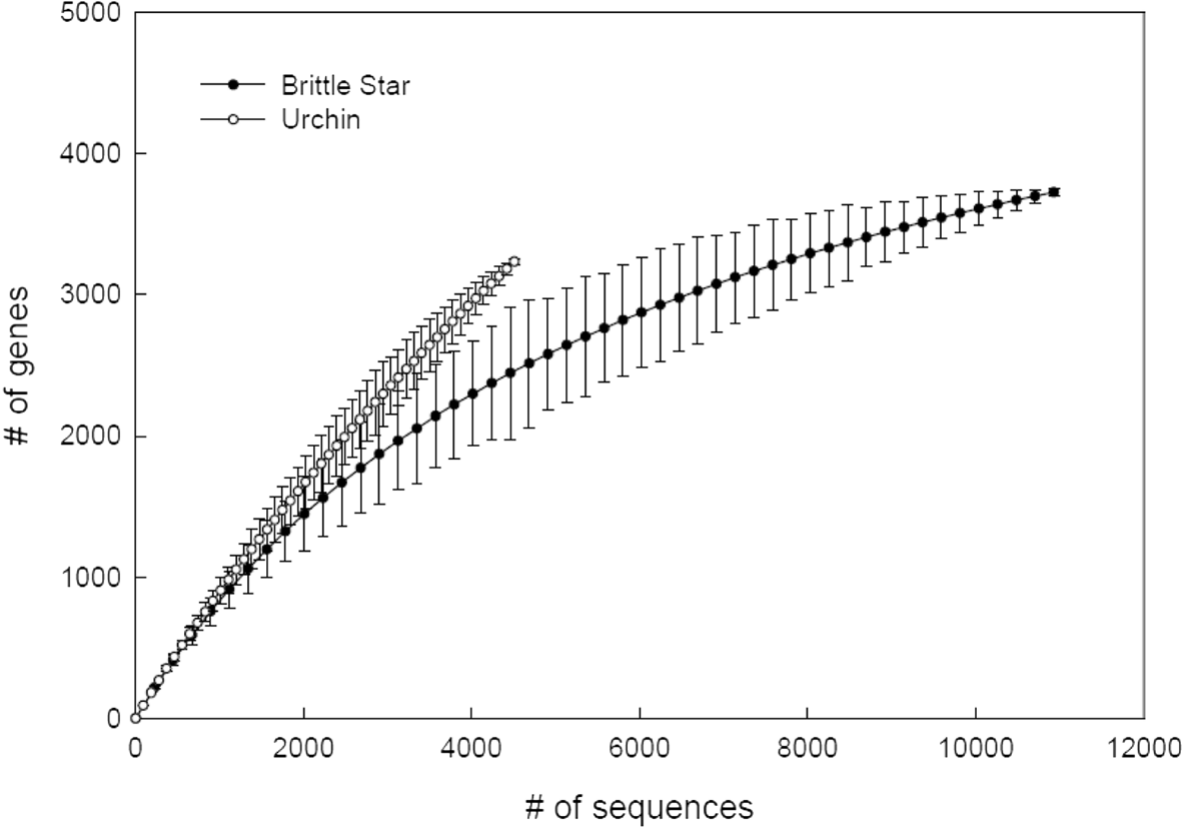
**Rarefaction curves for sea urchin and brittle star.** The steeper initial slope for the sea urchin curve indicates matches to a significant number of KEGG clusters even with many fewer sequences. The brittle star curve rises more gradually, but becomes asymptotic at the right, indicating that the sequencing captured most of the genes present in the gastrula transcriptome. If the two organisms express roughly the same number of genes at equivalent developmental stages, then the rarefaction curves indicate that comparison of these two data sets is indeed meaningful.

Note that the *O. wendtii* data were compared against data in each of the other datasets in Figure 5 separately. Therefore, brittle star sequences with hits to multiple datasets do not necessarily represent RBB matches between every component in the annotation, but merely represent significant hits between brittle star and more than one of the other datasets independently. Examination of the results reveals that the individual hits are mutually consistent in terms of genes identified. The brittle star data set has many times more sequences than the sea urchin gastrula UniGene set available on the NCBI UniGene database, and the average length of the brittle star sequences is shorter. To assess whether we could make meaningful comparisons between these different data sets, we plotted the data as rarefaction curves. In ecology, rarefaction uses repeated random re-sampling of a large pool of samples to estimate the species richness as a function of the number of individuals sampled. Here we used it to estimate how thoroughly each data set represents the full transcriptome. In Figure 6, the curve for sea urchin has a much steeper initial slope, and therefore matches to a significant number of KEGG clusters even with many fewer sequences. This likely occurs because the sea urchin sequences are longer on average. The brittle star curve rises more gradually, but plateaus near the end, indicating that the sequencing captured most of the genes present in the transcriptome. If we assume that the two organisms express roughly the same number of genes at equivalent developmental stages, then the rarefaction curves indicate that this is indeed a meaningful comparison. Comparison of the brittle star gastrula transcriptome to the *S. purpuratus* genome as well as to the *S. purpuratus* gastrula transciptome and to the KEGG Orthology database, allowed us to identify 3,385 sequence matches.

### Gene functional classes

*O. wendtii* sequences were compared to the KEGG Orthology database [24] by reciprocal best BLAST (Figure 3A). The KEGG Orthology database contains clusters of genes orthologous among a large number of organisms. Of the 3,800 clusters relevant to animals, 1,368 (36%) had significant matches to brittle star. Similarly, 1,335 KEGG clusters (35%) had matches to sea urchin gastrula. These numbers include 840 KEGG clusters (22%) with matches to both organisms.

When sorted into functional classes (Figure 3B), an average of 43%, 39%, and 28% of the distinct KEGG clusters within each class had matches to brittle star, to sea urchin, and to both, respectively, with a range between 2% and 85%. Each KEGG functional class consists of a number of biochemical pathways. On average, 44%, 43%, and 30% of the KEGG clusters within each pathway had matches to brittle star, to sea urchin, and to both, respectively. Note that there is extensive overlap between the various KEGG functional classes and pathways, with many clusters falling into several different ones.

Overall, genes involved in metabolism and genetic information processing were the most highly conserved, as would be expected. The number of these ‘housekeeping’ genes found in sea urchins and brittle stars are similar, and the relationship between the number of genes observed in each group and the number shared between them is very consistent. There are fewer orthologs detected in the other KEGG orthology groups. Many pathways under ‘Organ Systems’ and ‘Human Diseases’ are vertebrate-specific and/or relate to functions which do not operate extensively until later stages of development or after metamorphosis and would not be expected to be expressed at the gastrula stage. This is true for both organisms. There is also more variation in the number of gene matches to sea urchins and brittle stars in these functional classes.

Genes involved with the cytoskeleton and cell junctions had considerably more matches to brittle star. Cell-adhesion genes are often large, with many exons, and with domains often repeated and shared between multiple genes [36]. These characteristics, along with the short lengths of the brittle star sequences, have the potential to produce an artificially high number of BLAST hits. However, this pattern was the exception, not the rule, across the other functional classes.

The sea urchin had a far greater number of matches to genes involved in endocytosis, lysosome and RNA degradation. Many of these genes again overlap with several other pathways, but there is no clear pattern to account for the disparity.

### Comparison to sea urchin developmental gene regulatory network

The gene regulatory networks that underlie the differentiation of the basic tissue types in sea urchin embryos have been fairly well-characterized. The temporal and spatial expression of these genes has been determined and many of the regulatory interactions between the various genes have been determined, either directly or indirectly by interference with gene expression. The majority of these genes are expressed concurrently at the gastrula stage, which makes this stage an excellent point to identify a global set of genes important to the process of early cell differentiation. Here we use the sea urchin *S. purpuratus* gastrula GRNs at 21 to 30 h of development [3-6] as a reference to look for conservation of genes expressed in the brittle star gastrula at 40 h of development, which is equivalent morphologically. At this point the skeletal spicules have just begun forming, the archenteron is one third to halfway across the blastocoel cavity and the equivalent of secondary mesenchyme has formed. The gut is not yet partitioned and no mouth has formed. The presence of the same genes expressed at the same stage in these two organisms would suggest a conservation of GRNs and a shared gastrula ‘toolkit’ of proteins. The absence of genes expressed in either organism would indicate that there is either a temporal change in expression or that the gene is not expressed at all in the embryo of one group. Either is an indication of a change in a GRN. It is possible we could fail to detect some of those transcripts that are expressed at very low levels and recalcitrant to this RNA profiling. However, reciprocal BLAST searches using the brittle star gastrula transcriptome data and the *S. purpuratus* genome found homologs for a majority of genes involved in the sea urchin developmental gene regulatory network, including transcripts expressed at very low levels in *S. purpuratus*.

In sea urchins, a gradient of β-catenin initiated at the vegetal pole of the egg sets up and is soon reinforced by a circuit in the early embryo involving β–catenin/*lef1, wnt8, blimp1,* and *otx* in an intricate shifting relationship, creating a ring of gene expression which moves outward from the vegetal pole to specify endomesoderm [37]. *Hox11/13b* is also soon involved in this circuit [5,38]. Comparisons between sea urchins and starfish have revealed that just downstream from these early endomesoderm genes in the endoderm lies an extremely well-conserved kernel involving *blimp1/krox, otx, gatae, foxa,* and *brachyury*[39]. In starfish, *tbr (t-brain)* is also part of this kernel, a role which is likely deeply ancestral, as it is also expressed in vegetal pole endoderm precursors in both sea cucumbers and hemichordates [40,41]. However, in sea urchins *tbr* has lost this role in endoderm and has instead been co-opted into skeletogenesis [42]. In sand dollars it appears to play both these roles [43].

In Table 1 we compare some key endomesoderm and endoderm specific genes in the sea urchin to the transcripts present in the brittle star gastrula. Brittle stars express *β–catenin, lef1, otx, blimp1, wnt, hox/11/13band foxa* genes, suggesting that components of the endomesoderm and endoderm GRNs expressed early in development are conserved. *Gatae,* however, is not expressed. Many animal phyla employ *gata* genes in gut formation [44]. *Gatae* is a key component of the endoderm GRN in sea urchins and forms a feedback loop that maintains expression of these genes in the endoderm [45]. *Otx* and *blimp1* constitute another portion of that feedback loop [5], and this could be sufficient for endoderm differentiation in brittle stars. Two genes that are activated by *gatae* in *S. purpuratusbrachyury (bra)* and *krüppel (krl)*, are not expressed at the gastrula stage in brittle stars. *Krüppel* expression in sea urchins is highest in the early blastula, and is mostly gone by the time of gastrulation in *S. purpuratus*[46]. Its absence from the brittle star data may therefore reflect a small shift in timing and/or low transcript abundance at the onset of gastrulation. *T-brain* is not expressed in the brittle star gastrula. This would seem to indicate that *tbr* expression is not required for skeletogenesis in brittle star embryos as it is in sea urchins, or for endoderm formation as in starfish.

**Table 1:** **Comparison of *Ophiocoma wendtii *gastrula transcripts to the *Strongylocentrotus purpuratus *endodermal and endomesodermal gene regulatory networks**

**Gene**	**Found in *O.w. *gastrula**	** RBB to*S.p. *Genome [SpBase:]**	**RBB to NCBI RefSeq Proteins [NCBI:]**	**Role in *S.p.***
β-Catenin	Y	β-Catenin	*S.p.* β-Catenin	Endoderm
		[SPU_004319]	[XP_786059.2]	
Otx	Y	Otx	*S.p.* Otx	Endoderm
		[SPU_010424]	[NP_999753.2]	
Wnt	Y	Wnt5	*S.k.* Wnt2	Endoderm
		[SPU_026277]	[NP_001158455.1]	
Blimp1	Y	Blimp1/Krox	*B.f.* Zn-finger	Endoderm
		[SPU_027235]	[XP_002587482.1]	
Hox11/13b	Y	Hox11/13b	*S.p.* Hox11/13b	Endoderm
		[SPU_002631]	[NP_999774.1]	
Bra	N			Endoderm
Krl	N			Endoderm
Myc	Y	Myc	*S.p.* Myc	Endoderm
		[SPU_003166]	[NP_999744.1]	
SoxB1	Y	SoxB1	*S.p.* SoxB1	Endoderm
		[SPU_022820]	[NP_999639.1]	
Brn1-2-4	Y	Brn1-2-4	*S.p.* Brn1-2-4	Endoderm
		[SPU_016443]	[XP_782909.2]	
Tgif	Y	Tgif	*I.s.* Tgif	Endoderm
		[SPU_018126]	[XP_002433653.1]	
Hnf1	N			Endoderm
Eve	N			Endoderm
Hh	N			Endoderm
VEGF	Y	VEGF	*H.p.* VEGF	Endoderm
		[SPU_030148]	BAI67115.1]	
Dac	Y	Dac	*I.s.* Dachsund	Endoderm
		[SPU_028061]	[XP_002407755.1]	
Endo16	N			Endoderm
FoxA	Y	FoxA	*S.p.* FoxA	Endo + SMC
		[SPU_006676]	[NP_001073010.1]	
GataE	N			Endo + SMC
Kakapo	Y	Syne1	*S.p.* Similar to CG33715-PD	Endo + SMC
		[SPU_013237]	[XP_784190.2]	
Apobec	Y	Hnrpr	*S.p.* Hnrpr	Endo + SMC
		[SPU_019557]	[XP_793277.1]	
Gelsolin	Y	Gelsolin	*S.p.* Gelsolin	Endo + SMC
		[SPU_003985]	[XP_788777.1]	

B.f. = Branchiostoma floridae, H.p. = Heliocentrotus pulcherrimus.

I.s. = Ixodes scapularis, O.w. = Ophiocoma wendtii.

S.k. = Saccoglossus kowalevskii, S.p. = Strongylocentrotus purpuratus.

The endoderm in *S. purpuratus* is derived from two tiers of blastomeres formed during cleavage from the macromeres: Veg2, closest to the vegetal pole, and Veg1 above that. The Veg2 derived endoderm in *S. purpuratus* expresses *myc, brn1/2/4, tgif and dac* genes at the gastrula stage [47]. All of these are expressed in the brittle star gastrula (Table 1). In contrast, the Veg1 genes *eve* and *hnf1* are not expressed in brittle stars. Together this suggests that a central early kernel of the endoderm GRN is conserved, although the expression of *gatae* and some genes it regulates are not. The expression of genes found in Veg2 endoderm is also largely conserved. The most likely explanation of our results is that the equivalent of Veg1 endoderm has not formed in the brittle star gastrula at the stage we examined. This suggests a heterochronic shift in the formation of the second tier of endoderm. This could also explain the absence of *brachyury*. It is a key player in gut formation in both protostomes and deuterostomes, though the details differ between taxa [48-51]. A shift in the timing of Veg1 endoderm formation could delay expression of *brachyury* in the brittle star. A less likely explanation is that a loss of this layer of endoderm has occurred in brittle stars, and that the gut is formed entirely by the equivalent of Veg2 endoderm. *Endo16*, one of the major differentiation gene products in endoderm is not expressed in brittle star gastrula.

Following endomesoderm specification, mesenchyme precursors all express *ets1/2, erg, and hex* in *S. purpuratus.* All three of these genes are expressed in the brittle star gastrula (Table 2). The sea urchin skeletogenic primary mesenchyme derived from the micromeres, the homologous vegetal plate mesoderm in starfish, and the larval structures that produce the adult skeletons in both animals all express many of the same genes as sea urchin micromeres [19,52,53], and a majority of these genes were found in the brittle star gastrula transcriptome as well (Table 2). In all cases of echinoderm skeleton formation studied, including brittle star embryos, *alx1* is expressed. This is consistent with reports that ectopic expression of *alx1* in sea urchin non-skeletogenic mesenchyme (NSM) induces skeleton formation [52]. *Ets1* is expressed both maternally and zygotically, and is involved in all the above cases, activating a great number of downstream genes. *Ets1* and *alx1* were both found in the brittle star gastrula transcriptome. Just downstream from these in both sea urchin micromere and starfish vegetal plate mesoderm are a group of three genes, *erg, hex,* and *tgif,* which form a “lockdown” mechanism, stabilizing the specification state by feeding back to each other and to *tbr* and *ets1*, and feeding forward into tissue- specific differentiation genes [53]. All three were present in brittle star, as was *deadringer (dri)*, which appears to play a similar role in all the skeletogenic cases. *Tbr* has not been found in brittle stars. In starfish, *tbr* is seen in both endoderm (discussed above) and mesoderm [53]. It does not appear to be involved in adult skeletogenesis in either starfish or sea urchins. Its absence in brittle stars reinforces the idea that it was not part of the ancestral skeletal GRN and that its role in sea urchin embryonic skeleton formation is derived.

**Table 2:** **Comparison of *Ophiocoma wendtii *gastrula transcripts to the *Strongylocentrotus purpuratus *mesenchymal gene regulatory networks**

**Gene**	**Found in *O.w. *gastrula**	**RBB to *S.p. *Genome [SpBase:]**	**RBB to NCBI RefSeq Proteins [NCBI:]**	**Role in *S.p.***
HesC	Y	HesC	*S.p.* HesC	Mesenchyme
		[SPU_021608]	[XP_796692.1]	
Erg	Y	Erg	*S.p.* Erg	Mesenchyme
		[SPU_018483]	[NP_999833.1]	
Hex	Y	Hex	*S.p.* Hex	Mesenchyme
		[SPU_027215]	[XP_001197103.1]	
Ets1/2	Y	Ets1/2	*S.p.* Ets1/2	Mesenchyme
		[SPU_002874]	[NP_999698.1]	
Alx1	Y	Alx1	*S.p.* Alx1	PMC
		[SPU_025302]	[NP_999809.1]	
Tbr	N			PMC
Tgif	Y	Tgif	*I.s.* Tgif	PMC
		[SPU_18126]	[XP_002433653.1]	
FoxN2/3	N			PMC
Dri	Y	Dri	*S.p.* Dri	PMC
		[SPU_017106]	[NP_999799.1]	
FoxB	Y	FoxB	*S.p.* FoxB	PMC
		[SPU_004551]	[NP_999797.1]	
FoxO	Y	FoxO	*S.p.* FoxO	PMC
		[SPU_009178]	[XP_001183650.1]	
VEGFR	N			PMC
Delta	N			PMC
Spicule matrix genes	Possible	C-lectin [SPU_007882]	*S.p.* C-lectin [NP_999805.1]	Skeletal differentiation
MSP130	N			Skeletal differentiation
G-Cadherin	Y	G-Cadherin	*S.k.* G-Cadherin	Skeletal differentiation
		[SPU_015960]	[XP_002741140.1]	
Ficolin	Y	Fic	*B.f.* Ficolin	Skeletal differentiation
		[SPU_023548]	[XP_002594892.1]	
Cyclophilin	Y	CypL7	*D.m.* Cyclophilin 1	Skeletal differentiation
		[SPU_008305]	[NP_523366.2]	
Gcm	Y	Gcm	*S.k.* Gcm	SMC
		[SPU_006462]	[XP_002733441.1]	
Notch	N			SMC
Six1/2	N			SMC
Hnf6	Y	Hnf6	*S.p.* Hnf6	SMC
		[SPU_016449]	[NP_999824.1]	
GataC	N			SMC
Scl	N			SMC
Pks	Y	Pks	*S.p.* Pks	SMC
		[SPU_028395]	[NP_001239013.1]	
FoxF	Y	FoxF	*S.p.* FoxF	Small micromeres
		[SPU_000975]	[XP_794135.1]	
SoxE	N			Small micromeres
FoxY	N			Small micromeres

B.f. = Branchiostoma floridae, D.m. = Drosophila melanogaster, I.s. = Ixodes scapularis.

O.w. = Ophiocoma wendtii, S.k. = Saccoglossus kowalevskii.

S.p. = Strongylocentrotus purpuratus.

The downstream differentiation genes found in *S. purpuratus* skeletogenic cells at the gastrula stage are also found in the brittle star gastrula (Table 2). The spicule matrix proteins of the sea urchin endoskeleton contain a single C-type lectin domain and repetitious stretches rich in proline and glycine [54-56]. The apparently loose constraints on primary structure in these proteins, and the resulting low sequence conservation make identification of brittle star homologs difficult. However, the brittle star gastrula transcriptome contains several transcripts encoding C-type lectin domains and repetitive regions. Several other proteins, including Cyclophilin and Ficolin, are all expressed in sea urchin PMC cells and associated with the skeleton, though their exact functions remain unclear. The brittle star gastrula transcriptome contains matches for *Cyclophilin* and F*icolin*, but not for *MSP130*, a major cell surface protein in sea urchin PMCs. Overall there is a remarkable conservation of the GRN leading to formation of mineralized tissue in the embryos of sea urchins and brittle stars.

In sea urchins, Delta-Notch signaling from the micromeres activates *gcm* in the adjacent NSM to form pigment cells [57,58]. Brittle star embryos do not form embryonic pigment cells. Neither do starfish, but they express *gcm* in ectoderm rather than mesoderm, and it does not depend on Delta signaling [18]. Neither *notch* nor *delta* is expressed in the brittle star gastrula (Table 2). *Gcm* is expressed in brittle star gastrula, but *gatac, gatae*. *six1/2,*and *scl* are not. This suggests that the GRN leading to pigment cells, not surprisingly, is not conserved in brittle stars. Likewise, most of the genes that are expressed in the *S. purpuratus* small micromeres (i.e. *soxe, foxy*), which are not formed outside of euechinoids, are not expressed in the brittle star gastrula (Table 2).

In sea urchin ectoderm, Nodal patterns both the ventro-dorsal (oral-aboral) and left-right axes [59], but was not found to be transcribed in brittle star gastrula (Table 3); nor was its antagonist Lefty, which limits Nodal to the ventral side during sea urchin development [60]. On the other hand, a number of genes downstream from Nodal and key to specification of different ectodermal regions [61] were found in brittle star (Table 3). Most of the genes expressed in the *S. purpuratus* oral ectoderm are found in the brittle star gastrula transcriptome, including *chordin* and *BMP2/4*. Sea urchin BMP2/4 is expressed in the oral ectoderm, then diffuses to and specifies the aboral ectoderm by inhibiting Nodal [62], while Chordin helps pattern neural tissue in the ciliary band at the oral/aboral border by excluding BMP2/4 activity from the oral side [63]. Genes that, in the sea urchin, are activated by Nodal-independent early oral ectoderm input are found to be expressed in brittle star gastrula. These include *otxb1/2* and *hnf6.* Of the sea urchin genes that are activated at the boundary of ectoderm and endoderm, *foxj* is expressed in brittle star gastrula, but *lim1* and *nk1* are not.

**Table 3:** **Comparison of *Ophiocoma wendtii *gastrula transcripts to the *Strongylocentrotus purpuratus *ectodermal gene regulatory network**

**Gene**	**Found in*O.w.*gastrula**	**RBB to*S.p.*Genome [SpBase:]**	**RBB to NCBI RefSeq Proteins [NCBI:]**	**Role in *S.p.***
Nodal	N			Oral ectoderm
Lefty	N			Oral ectoderm
Chordin	Y	Chordin	*S.k.* Chordin	Oral ectoderm
		[SPU_004983]	[NP_001158390.1]	
Sip1	N			Oral ectoderm
FoxG	N			Oral ectoderm
BMP2/4	Y	BMP2/4	*S.p.* BMP2/4	Oral ectoderm
		[SPU_000669]	[NP_001116977.1]	
FoxA	Y	FoxA	*S.p.* FoxA	Oral ectoderm
		[SPU_006676]	[NP_001073010.1]	
Bra	N			Oral ectoderm
Dri	Y	Dri	*S.p.* Dri	Oral ectoderm
		[SPU_017106]	[NP_999799.1]	
Hes	Y	Hes	*S.k.* Hes1	Oral ectoderm
		[SPU_006814]	[NP_001158466.1]	
Hnf6	Y	Hnf6	*S.p.* Hnf6	Oral ectoderm
		[SPU_016449]	[NP_999824.1]	
FoxJ1	Y	FoxJ1	*S.p.* FoxJ1	Ecto/Endo border
		[SPU_027969]	[NP_001073013.1]	
Nk1	N			Ecto/Endo border
Lim1	N			Ecto/Endo border
Tbx2/3	Y	Tbx2/3	*S.p.* Tbx2/3	Aboral ectoderm
		[SPU_023386]	[NP_001123280.1]	
Lhx2 (Lim2)	Y	Lhx2	*M.m.* Lhx2	Aboral ectoderm
		[SPU_021313]	[NP_034840.1]	
Dlx	N			Aboral ectoderm
Nk2.2	Y	Nk2.2	*S.p.* Nk2.2	Aboral ectoderm
		[SPU_000756]	[NP_001123283.1]	
Hox7	N			Aboral ectoderm
Msx	N			Aboral ectoderm
Klf7	Y	Klf2/4	*S.k.* Klf2	Aboral ectoderm
		[SPU_020311]	[NP_001161575.1]	
IrxA	N			Aboral ectoderm
Hmx	N			Aboral ectoderm

*M.m. = Mus musculus, O.w. = Ophiocoma wendtii,S.k. = Saccoglossus kowalevskii, S.p. = Strongylocentrotus purpuratus*.

Genes that are expressed in the sea urchin aboral ectoderm are not as uniformly expressed in brittle star gastrula. Genes expressed by 12 h of sea urchin development such as *sim* and *nk2.2* are expressed in brittle star gastrula, but not genes expressed later in sea urchin aboral ectoderm such as *hox7* and *msx*. The differentiation genes *spec1* and *spec2a* are also not found to be expressed in brittle star gastrula at the time examined. *Tbx2/3* is expressed in brittle star gastrula, but not *irxa* and *dlx*, which are activated by Tbx2/3 in sea urchins. Taken together this would suggest two heterochronic shifts in ectoderm determination between sea urchins and brittle stars. In sea urchins, all of the genes examined are expressed at the gastrula stage. In brittle stars, patterning by Nodal and Lefty is apparently complete by gastrulation and these genes are no longer expressed. Oral ectoderm is determined and specification of the aboral ectoderm is underway, but it appears that this process is not complete in the 40 h brittle star gastrula.

## Conclusions

The brittle star *O. wendtii* exhibits radial holoblastic cleavages that are equal throughout, giving rise to uniform-sized blastomeres without the formation of the micromeres characteristic to sea urchins. Despite this, mesenchymal cells ingress and give rise to an embryonic skeleton, a developmental structure unique to echinoids and ophiuroids among the echinoderms. Mesenchymal cells also give rise to the coelomic pouches, but no pigment cells are formed in the embryo. Archenteron formation occurs much the same as in sea urchins, although there is a delay in gut elongation following invagination as well as in growth of the skeletal spicules initiated in the ventrolateral clusters. The resulting pluteus larva closely resembles that of sea urchins, albeit without pigment cells. The *O. wendtii* gastrula expresses genes from all functional classes at the gastrula stage. Brittle stars and sea urchins have comparable numbers of genes in most functional classes expressed at the gastrula stage.

A majority of the genes involved in the sea urchin gene regulatory network were also found in the brittle star gastrula transcriptome (Table 4). The brittle star pyrosequencing data are completely consistent with our earlier results using a PCR-based candidate gene approach (not shown). For example, transcripts of *alx1, dri, gabp, ets1*, and *erg* were found by both methods, whereas *tbr, gatac,* and *gatae* were not. While this does not completely rule out these genes being expressed but undetected by our methods, their absence is striking given the overall conservation of expression between the two groups. The percentage of genes involved in gene regulatory networks expressed in *S. purpuratus* gastrula that are also expressed in *O. wendtii* gastrula exceeds the percentage of transcripts conserved overall (Table 4). However, this conservation is not uniform across the different tissue types found in echinoderm gastrulae. Some of these differences can be explained by heterochronic shifts in gene expression, although loss of gene expression is also a possibility. Some of the endomesoderm genes that are expressed in sea urchin gastrula at declining levels could be undetectable by the brittle star gastrula stage. Examination of the aboral ectoderm genes expressed in *O. wendtii* relative to *S. purpuratus* indicates that specification of aboral ectoderm has begun but is delayed in the brittle star. The same could be true for the Veg1 endoderm. Other differences in gene expression correlate with differences in embryonic development. Brittle star embryos do not possess micromeres or pigment cells. The second lowest percentage of GRN genes conserved (33%) is seen in the genes expressed in *S. purpuratus* small micromeres and pigment cells (Table 4).

**Table 4:** **Conservation of genes between *Strongylocentrotus purpuratus *and *Ophiocoma wendtii***

		**% conserved in *O. wendtii***
*S. purpuratus* transcriptome		55
Early gastrula GRN		65
Endoderm		53
	Veg2 endoderm	70
	Veg1 endoderm	20
Primary mesenchyme		86
Non-skeletogenic mesenchyme		57
	Secondary mesenchyme	64
	Small micromeres	33
Oral ectoderm		67
Aboral ectoderm		58

The highest percentage of GRN conservation is seen in the skeletogenic mesenchyme cells (PMCs in sea urchins). This is not surprising, since all adult echinoderms form mineralized structures. The GRN and differentiation genes that lead to mineralized structures must be conserved in order for the adult skeleton to form. In sea urchins this GRN is activated in the embryo largely intact. The conservation of these genes in the *O. wendtii* gastrula suggests that is the case in brittle stars as well. An extensive analysis of spatial expression of the genes involved in these GRNs is the next step in confirmation of homology.

In sea urchins, Hesc is a transcriptional repressor ubiquitously expressed in the embryo, where its role is to repress the skeleton program. In the sea urchin micromeres, *hesc* is itself repressed by Pmar1 in response to nuclearized β-catenin, thereby de-repressing the skeleton circuits [64]. This double-negative *pmar1/hesc* gate appears unique to sea urchins as the mechanism that coupled the pre-existing programs of skeletogenesis and maternal β-catenin-mediated vegetal specification to produce the novelty of the embryonic skeleton, as it is not involved in adult sea urchin skeletogenesis [19,53]. Recent evidence suggests that other, as yet unknown, mechanisms related to the unequal cleavage that produces the micromeres are also involved [65]. Starfish, which do not build an embryonic skeleton, also express *hesc* throughout most of the embryo, but this expression appears to have no effect on mesodermal genes shared with sea urchin skeletogenesis, and *pmar1* has never been found in starfish [54].

Sea urchins express *pmar1* from fourth cleavage through mid-blastula, so we would not expect to see it expressed in the *O. wendtii* gastrula transcriptome. Using PCR, we have searched for, but never found, *pmar1* transcripts from any stage of brittle star development. We have, however, successfully amplified the *pmar1* homolog from brittle star genomic DNA, identified as such by the presence of a conserved intron [unpublished]. This suggests that activation of the adult skeletal GRN in embryos occurred differently in brittle stars than in sea urchins. Overall, our data suggest that embryonic skeleton formation in sea urchins and brittle stars represents convergent evolution by independent co-optation of a shared pathway utilized in adult skeleton formation.

## Abbreviations

BLAST: Basic Local Alignment Search Tool; bp: Base pairs; EST: Expressed sequence tag; GRN: Gene regulatory network; NSM: Non-skeletogenic mesenchyme; PMC: Primary mesenchyme; RBB: Reciprocal best BLAST; SMC: Secondary mesenchyme.

## Competing interests

The authors declare that they have no competing interests.

## Authors’ contributions

BTL conceived of and oversaw the project, collected animals, cultured embryos, extracted RNA, and conducted BLAST comparisons against the sea urchin GRN. NG performed, and WKT oversaw and advised on, initial processing of the data and automated BLAST searches. RV assembled the annotation data, carried out the analyses and interpretation, and wrote most of the manuscript. JRG oversaw and advised on the analyses. All authors contributed to the final version of the manuscript.

## References

[B1] Davidson EH Rast JP Oliveri P Ransick A Calestani C Yuh CH Minokawa T Amore G Hinman V Arenas-Mena C Otim O Brown CT Livi CB Lee PY Revilla R Rust AG Pan Z Schilstra MJ Clarke PJ Arnone MI Rowen L Cameron RA McClay DR Hood L Bolouri H A genomic regulatory network for development Science 2002 295 1669 1678 10.1126/science.1069883 11872831

[B2] Davidson EH Rast JP Oliveri P Ransick A Calestani C Yuh CH Minokawa T Amore G Hinman V Arenas-Mena C Otim O Brown CT Livi CB Lee PY Revilla R Schilstra MJ Clarke PJ Rust AG Pan Z Arnone MI Rowen L Cameron RA McClay DR Hood L Bolouri H A provisional regulatory gene network for specification of endomesoderm in the sea urchin embryo Dev Biol 2002 246 162 190 10.1006/dbio.2002.0635 12027441

[B3] Oliveri P Davidson EH Gene regulatory network controlling embryonic specification in the sea urchin Curr Opin Genet Dev 2004 204 351 380 15261650 10.1016/j.gde.2004.06.004

[B4] Su YH Gene regulatory networks for ectoderm specification in sea urchin embryos Biochim Biophys Acta 2009 1789 261 267 10.1016/j.bbagrm.2009.02.002 19429544

[B5] Peter IS Davidson EH The endoderm gene regulatory network in sea urchin embryos up to mid-blastula stage Dev Biol 2010 340 188 199 10.1016/j.ydbio.2009.10.037 19895806 PMC3981691

[B6] Cameron RA Samanta M Yuan A He D Davidson E SpBase: the sea urchin genome database and web site Nucleic Acids Res 2009 37 D750 D754 http://spbase.org 10.1093/nar/gkn887 19010966 PMC2686435

[B7] Sea Urchin Genome Sequencing Consortium The genome of the sea urchin Strongylocentrotus purpuratus Science 2006 314 941 952 17095691 10.1126/science.1133609 PMC3159423

[B8] Poustka AJ Herwig R Krause A Hennig S Meier-Ewert S Lehrach H Toward the gene catalogue of sea urchin development: the construction and analysis of an unfertilized egg cDNA library highly normalized by oligonucleotide fingerprinting Genomics 1999 59 122 133 10.1006/geno.1999.5852 10409423

[B9] Lee YH Huang GM Cameron RA Graham G Davidson EH Hood L Britten RJ EST analysis of gene expression in early cleavage-stage sea urchin embryos Development 1999 126 3857 3867 10433914 10.1242/dev.126.17.3857

[B10] Zhu X Mahairas G Illies M Cameron RA Davidson EH Ettensohn CA A large-scale analysis of mRNAs expressed by primary mesenchyme cells of the sea urchin embryo Development 2001 128 2615 2627 11493577 10.1242/dev.128.13.2615

[B11] Poustka AJ Groth D Hennig S Thamm S Cameron A Beck A Reinhardt R Herwig R Panopoulou G Lehrach H Generation, annotation, evolutionary analysis, and database integration of 20,000 unique sea urchin EST clusters Genome Res 2003 13 2736 2746 10.1101/gr.1674103 14656975 PMC403816

[B12] Wei Z Angerer RC Angerer LM A database of mRNA expression patterns for the sea urchin embryo Dev Biol 2006 300 476 484 10.1016/j.ydbio.2006.08.034 17007833 PMC1762123

[B13] Materna SC Nam J Davidson EH High accuracy, high-resolution prevalence measurement for the majority of locally expressed regulatory genes in early sea urchin development Gene Expr Patterns 2010 10 177 184 10.1016/j.gep.2010.04.002 20398801 PMC2902461

[B14] Paul CRC Smith AB The early radiation and phylogeny of echinoderms Biol Rev 1984 59 443 481 10.1111/j.1469-185X.1984.tb00411.x

[B15] Littlewood DTJ Smith AB Clough KA Emson RH The interrelationships of the echinoderm classes: morphological and molecular evidence Biol J Linnean Soc 1997 61 409 438 10.1111/j.1095-8312.1997.tb01799.x

[B16] Harmon MC The position of the ophiuroidea within the phylum echinodermata 2005 University of South Florida, Biology Department

[B17] Davidson EH Erwin DH Gene regulatory networks and the evolution of animal body plans Science 2006 311 796 800 10.1126/science.1113832 16469913

[B18] Hinman VF Davidson EH Evolutionary plasticity of developmental gene regulatory network architecture Proc Natl Acad Sci USA 2007 104 19404 19409 10.1073/pnas.0709994104 18042699 PMC2148302

[B19] Gao F Davidson EH Transfer of a large gene regulatory apparatus to a new developmental address in echinoid evolution Proc Natl Acad Sci USA 2008 105 6091 6096 10.1073/pnas.0801201105 18413604 PMC2329712

[B20] Wilt FH Ettensohn CA Bauerlin E Handbook of Biomineralization The morphogenesis and biomineralization of the sea urchin larval skeleton 2007 Weinheim Germany: Wiley-VCH 183 210

[B21] Ettensohn CA Lessons from a gene regulatory network: echinoderm skeletogenesis provides insights into evolution, plasticity and morphogenesis Development 2009 136 11 21 10.1242/dev.023564 19060330

[B22] Wray GA McClay DR The origin of spicule-forming cells in a “primitive” sea urchin (Eucidares tribloides) which appears to lack primary mesenchyme cells Development 1988 103 305 315 3066611 10.1242/dev.103.2.305

[B23] Meyer E Aglyamova GV Wang S Buchanan-Carter J Abrego D Colbourne JK Willis BL Matz MV Sequencing and de novo analysis of a coral larval transcriptome using 454 GSFlx BMC Genomics 2009 10 219 10.1186/1471-2164-10-219 19435504 PMC2689275

[B24] Altschul SF Gish W Miller W Myers EW Lipman DJ Basic local alignment search tool J Mol Biol 1990 215 403 410 2231712 10.1016/S0022-2836(05)80360-2

[B25] Lerat E Daubin V Ochman H Moran NA Evolutionary origins of genomic repertoires in bacteria PLoS Biol 2005 3 e130 10.1371/journal.pbio.0030130 15799709 PMC1073693

[B26] Cooper VS Vohr SH Wrocklage SC Hatcher PJ Why genes evolve faster on secondary chromosomes in bacteria PLoS Comput Biol 2010 6 e1000732 10.1371/journal.pcbi.1000732 20369015 PMC2848543

[B27] Flynn KM Vohr SH Hatcher PJ Cooper VS Evolutionary rates and gene dispensability associate with replication timing in the archaeon Sulfolobus islandicus Genome Biol Evol 2010 2 859 869 10.1093/gbe/evq068 20978102 PMC3000693

[B28] Garnhart N Bergeron RD Homology Inspector (HomIn): A Tool for Exploring Homologyhttp://www.cs.unh.edu/~rdb/reports/homin.pdf

[B29] Kanehisam K Goto S KEGG: Kyoto encyclopedia of genes and genomes Nucleic Acids Res 2000 28 27 30 10.1093/nar/28.1.27 10592173 PMC102409

[B30] Tatusov RL Koonin EV Lipman DJ A genomic perspective on protein families Science 1997 278 631 637 10.1126/science.278.5338.631 9381173

[B31] Tatusov RL Fedorova ND Jackson JD Jacobs AR Kiryutin B Koonin EV Krylov DM Mazumder R Mekhedov SL Nikolskaya AN Rao BS Smirnov S Sverdlov AV Vasudevan S Wolf YI Yin JJ Natale DA The COG database: an updated version includes eukaryotes BMC Bioinforma 2003 4 41 10.1186/1471-2105-4-41 PMC222959 12969510

[B32] Ashburner M Ball CA Blake JA Botstein D Butler H Cherry JM Davis AP Dolinski K Dwight SS Eppig JT Harris MA Hill DP Issel-Tarver L Kasarskis A Lewis S Matese JC Richardson JE Ringwald M Rubin GM Sherlock G Gene ontology: tool for the unification of biology. The gene ontology consortium Nat Genet 2000 25 25 29 10.1038/75556 10802651 PMC3037419

[B33] EcoSim: null models software for ecology Version 7. 2011 http://garyentsminger.com/ecosim.htm

[B34] UniGene sea urchin embryo cDNA libraries http://www.ncbi.nlm.nih.gov/UniGene/lbrowse2.cgi?TAXID=7668&CUTOF F=1000

[B35] KEGG Orthology Databasehttp://www.genome.jp/kegg/ko.html

[B36] Whittaker CA Bergeron KF Whittle J Brandhorst BP Burke RD Hynes RO The echinoderm adhesome Dev Biol 2006 300 252 266 10.1016/j.ydbio.2006.07.044 16950242 PMC3565218

[B37] Smith J Theodoris C Davidson EH A gene regulatory network subcircuit drives a dynamic pattern of gene expression Science 2007 318 794 797 10.1126/science.1146524 17975065

[B38] Smith J Kraemer E Liu H Theodoris C Davidson E A spatially dynamic cohort of regulatory genes in the endomesodermal gene network of the sea urchin embryo Dev Biol 2008 313 863 875 10.1016/j.ydbio.2007.10.042 18061160 PMC3640430

[B39] Hinman VF Nguyen AT Cameron A Davidson EH Developmental gene regulatory network architecture across 500 million years of echinoderm evolution Proc Natl Acad Sci USA 2003 100 13356 13361 10.1073/pnas.2235868100 14595011 PMC263818

[B40] Maruyama YK A sea cucumber homolog of the mouse T-Brain-1 is expressed in the invaginated cells of the early gastrula in Holothuria leucospilota Zoolog Sci 2000 17 383 387 18494594 10.2108/jsz.17.383

[B41] Tagawa K Humphreys T Satoh N T-Brain expression in the apical organ of hemichordate tornaria larvae suggests its evolutionary link to the vertebrate forebrain J Exp Zool B Mol Dev Evol 2000 288 23 31 10.1002/(SICI)1097-010X(20000415)288:1<23::AID-JEZ3>3.0.CO;2-H 10750050

[B42] Hinman VF Nguyen A Davidson EH Caught in the evolutionary act: precise cis-regulatory basis of difference in the organization of gene networks of sea stars and sea urchins Dev Biol 2007 312 584 595 10.1016/j.ydbio.2007.09.006 17956756

[B43] Minemura K Yamaguchi M Minokawa T Evolutionary modification of T- brain (tbr) expression patterns in sand dollar Gene Expr Patterns 2009 9 468 474 10.1016/j.gep.2009.07.008 19635588

[B44] Patient RK McGhee JD The GATA family (vertebrates and invertebrates) Curr Opin Genet Dev 2002 12 416 422 10.1016/S0959-437X(02)00319-2 12100886

[B45] Yuh CW Dorman ER Howard ML Davidson EH An otx cis-regulatory module: a key node in the sea urchin endomesoderm gene regulatory network Dev Biol 2004 269 536 551 10.1016/j.ydbio.2004.02.025 15110718

[B46] Howard EW Newman LA Oleksyn DW Angerer RC Angerer LM SpKrl: a direct target of β-catenin regulation required for endoderm differentiation in sea urchin embryos Development 2001 128 365 375 11152635 10.1242/dev.128.3.365

[B47] Peter IS Davidson EH A gene regulatory network controlling the embryonic specification of endoderm Nature 2011 474 635 639 10.1038/nature10100 21623371 PMC3976212

[B48] Peterson KJ Harada Y Cameron RA Davidson EH Expression Pattern of Brachyury and Not in the Sea Urchin: Comparative Implications for the Origins of Mesoderm in the Basal Deuterostomes Dev Biol 1999 207 419 431 10.1006/dbio.1998.9177 10068473

[B49] Shoguchi E Satoh N Maruyama YK Pattern of Brachyury gene expression in starfish embryos resembles that of hemichordate embryos but not of sea urchin embryos Mech Dev 1999 82 185 189 10.1016/S0925-4773(99)00008-8 10354483

[B50] Mitsunaga-Nakatsubo K Harada Y Satoh N Shimada H Akasaka K Brachyury homolog (HpTa) is involved in the formation of archenteron and secondary mesenchyme cell differentiation in the sea urchin embryo Zoology 2001 104 99 102 10.1078/0944-2006-2410018 16351823

[B51] Gross JM McClay DR The role of Brachyury (T) during gastrulation movements in the sea urchin Lytechinus variegatus Dev Biol 2001 239 132 147 10.1006/dbio.2001.0426 11784024

[B52] Ettensohn CA Kitazawa C Cheers MS Leonard JD Sharma T Gene regulatory networks and developmental plasticity in the early sea urchin embryo: alternative deployment of the skeletogenic gene regulatory network Development 2007 134 3077 3087 10.1242/dev.009092 17670786

[B53] McCauley BS Weideman EP Hinman VF A conserved gene regulatory network subcircuit drives different developmental fates in the vegetal pole of highly divergent echinoderm embryos Dev Biol 2010 340 200 208 10.1016/j.ydbio.2009.11.020 19941847

[B54] Livingston BT Killian CE Wilt F Cameron A Landrum MJ Ermolaeva O Sapojnikov V Maglott DR Buchanan AM Ettensohn CA A genome-wide analysis of biomineralization-related proteins in the sea urchin Strongylocentrotus purpuratus Dev Biol 2006 300 335 348 10.1016/j.ydbio.2006.07.047 16987510

[B55] Mann K Poustka AJ Mann M The sea urchin (Strongylocentrotus purpuratus) test and spine proteomes Proteome Sci 2008 6 22 10.1186/1477-5956-6-22 18694502 PMC2527298

[B56] Mann K Wilt FH Poustka AJ Proteomic analysis of sea urchin (Strongylocentrotus purpuratus) spicule matrix Proteome Sci 2010 8 33 10.1186/1477-5956-8-33 20565753 PMC2909932

[B57] Ransick A Davidson EH cis-regulatory processing of Notch signaling input to the sea urchin glial cells missing gene during mesoderm specification Dev Biol 2006 297 587 602 10.1016/j.ydbio.2006.05.037 16925988

[B58] Croce JC McClay DR Dynamics of Delta/Notch signaling on endomesoderm segregation in the sea urchin embryo Development 2010 137 83 91 10.1242/dev.044149 20023163 PMC2796929

[B59] Duboc V Lepage T A conserved role for the nodal signaling pathway in the establishment of dorso-ventral and left–right axes in deuterostomes J Exp Zool B Mol Dev Evol 2008 310B 41 53 10.1002/jez.b.21121 16838294

[B60] Duboc V Lapraz F Besnardeau L Lepage T Lefty acts as an essential modulator of Nodal activity during sea urchin oral-aboral axis formation Dev Biol 2008 320 49 59 10.1016/j.ydbio.2008.04.012 18582858

[B61] Saudemont A Haillot E Mekpoh F Bessodes N Quirin M Lapraz F Duboc V Röttinger E Range R Oisel A Besnardeau L Wincker P Lepage T Ancestral regulatory circuits governing ectoderm patterning downstream of nodal and BMP2/4 revealed by gene regulatory network analysis in an Echinoderm PLoS Genet 2010 6 12 1 31 10.1371/journal.pgen.1001259 PMC3009687 21203442

[B62] Lapraz F Besnardeau L Lepage T Patterning of the dorsal-ventral axis in echinoderms: insights into the evolution of the BMP-chordin signaling network PLoS Biol 2009 7 e1000248 10.1371/journal.pbio.1000248 19956794 PMC2772021

[B63] Bradham CA Oikonomou C Kühn A Core AB Modell JW McClay DR Poustka AJ Chordin is required for neural but not axial development in sea urchin embryos Dev Biol 2009 328 221 233 10.1016/j.ydbio.2009.01.027 19389361 PMC2700341

[B64] Revilla-i-Domingo R Oliveri P Davidson EH A missing link in the sea urchin embryo gene regulatory network: hesC and the double-negative specification of micromeres Proc Natl Acad Sci USA 2007 104 12383 12388 10.1073/pnas.0705324104 17636127 PMC1941478

[B65] Sharma T Ettensohn CA Activation of the skeletogenic gene regulatory network in the early sea urchin embryo Development 2010 137 1149 1157 10.1242/dev.048652 20181745

